# Ether Irrigations in Strangulated Hernia

**Published:** 1890-02

**Authors:** 


### ETHER IRRIGATIONS IN STRANGULATED HERNIA.

Dr. Ivan N. Drakin (*The Annals of Surgery*, August, 1889) eulogizes ether irrigations as an exellent means for the reduction of strangulated hernia. He simply pours a teaspoonful of ether over the hernial tumor every quarter or half hour, keeping it covered with compresses during the intervals. As a rule, after three or four tablespoonfuls, the intestinal loop slips down into the abdominal cavity by itself; in some cases, however, slight pressure should be applied. In the case of incarcerated scrotal hernia, it is advisable to irrigate with a mixture of ether (twenty parts) and hyoscyamus oil four parts). When applied sufficiently early, the method is said to give most brilliant results.

Dr. A. J. Brustien (*Ibid,*) describes the case of a woman with an incarcerated umbilical hernia of the size of a man's fist, in whom, after all ordinary procedures for reduction had failed, irrigation with a small jet of ether was resorted to, taxis being continued at the same time. In three or four minutes the reduction was affected with striking ease. The action of local etherization was attributed to a rapid contraction of the intestinal wall, and to the diminution in volume of the hernial gaseous contents caused by the sudden lowering of temperature.—*Am. Journal Medical Sciences.*

